# Neurosurgical Approaches to Brain Tissue Harvesting for the Establishment of Cell Cultures in Neural Experimental Cell Models

**DOI:** 10.3390/ma14226857

**Published:** 2021-11-13

**Authors:** Lidija Gradišnik, Roman Bošnjak, Gorazd Bunc, Janez Ravnik, Tina Maver, Tomaž Velnar

**Affiliations:** 1 Faculty of Medicine, Institute of Biomedical Sciences, University of Maribor, Taborska 8, 2000 Maribor, Slovenia; lidija.gradisnik@um.si; 2 Alma Mater Europaea ECM, Slovenska 17, 2000 Maribor, Slovenia; 3 Department of Neurosurgery, University Medical Centre Ljubljana, Zaloska 7, 1000 Ljubljana, Slovenia; roman.bosnjak@kclj.si; 4 Department of Neurosurgery, University Medical Centre Maribor, Ljubljanska 5, 2000 Maribor, Slovenia; gorazd.bunc@ukc-mb.si (G.B.); janez.ravnik@ukc-mb.si (J.R.); 5 Department of Pharmacology, Faculty of Medicine, University of Maribor, Taborska ulica 8, 2000 Maribor, Slovenia

**Keywords:** cell isolation, brain, experimental cell models, biomaterial testing, neurosurgery

## Abstract

In recent decades, cell biology has made rapid progress. Cell isolation and cultivation techniques, supported by modern laboratory procedures and experimental capabilities, provide a wide range of opportunities for in vitro research to study physiological and pathophysiological processes in health and disease. They can also be used very efficiently for the analysis of biomaterials. Before a new biomaterial is ready for implantation into tissues and widespread use in clinical practice, it must be extensively tested. Experimental cell models, which are a suitable testing ground and the first line of empirical exploration of new biomaterials, must contain suitable cells that form the basis of biomaterial testing. To isolate a stable and suitable cell culture, many steps are required. The first and one of the most important steps is the collection of donor tissue, usually during a surgical procedure. Thus, the collection is the foundation for the success of cell isolation. This article explains the sources and neurosurgical procedures for obtaining brain tissue samples for cell isolation techniques, which are essential for biomaterial testing procedures.

## 1. Introduction

Cell biology has advanced rapidly in recent decades [1]. Novel laboratory procedures, methods, instruments, and experimental conditions offer a wide range of research possibilities on cell cultures and cell models, which are a suitable alternative to experiments on animals and humans under so-called in vivo conditions [2,3]. Cells isolated from tissues can be maintained in cell culture, transformed into cell lines, and included for experiments in various cell models, which are becoming increasingly important in the study of physiological and pathophysiological processes in vitro. The scope of application is extensive and includes the preclinical and clinical areas of medicine and other research areas such as experimental pharmacy and the pharmaceutical, cosmetic, and food industries [4,5,6].

There is no universal cell culture or cell line suitable for all experimental cell models [7,8], meaning that specific experiments can only be performed on particular cell types. Therefore, the isolation of new cell types from tissues and their propagation is of utmost importance [2,9]. The most suitable tissue for cell isolation is fresh donor tissue collected during various surgeries and immediately transferred to a cell laboratory where isolation is performed. The method and timing of tissue collection, storage during surgery and transport, and transfer speed to the laboratory all matter. Improper tissue collection and handling can damage the cells in the tissue sample [10,11,12]. Tissues have varying degrees of sensitivity to harvesting, storage, transport, and waiting for processing in the laboratory, affecting the yield of cell isolation and their growth in culture. Brain tissue, which is used as a source for various types of cell isolation, is susceptible. Both normal and tumorous brain tissue is vital for cell isolation and further experiments on isolated cells, each having its advantages and disadvantages [13,14,15,16]. 

A sample of brain tissue collected during various neurosurgical procedures can therefore be a good source for the isolation of neurons, astrocytes, oligodendroglia, microglia, ependymal and microvascular cells, and various neoplastic cells from brain tumours [17,18,19,20]. Astrocytes, oligodendroglial and microvascular cells of cerebral vessels, are involved in inflammatory and neurodegenerative and reparative mechanisms. They not only perform the supportive functions already mentioned: because of their role in neurotrauma, epilepsy, Alzheimer’s and Parkinson’s disease, multiple sclerosis, and various forms of dementia, their isolation and integration into experimental cell models are of utmost importance. Among tumours, gliomas of multiple grades are the most studied pathology and the primary source of tumour cell isolation [21,22,23].

Adult human brain tissue is an essential requirement for cell culture and for a variety of biotechnological tests used in preclinical practice. Successful isolation and preservation of isolated cells in vitro requires consideration of surgical technique of tissue collection, the surgical approach, the technique and manipulation with the tissue, its storage, the method, and the timing of tissue transfer to the laboratory. This is one of the fundamentals that determine the success of cell isolation. Not all surgical methods are equally suitable. Therefore, neurosurgical approaches to brain tissue sampling are of great importance for brain cell isolation techniques [24,25]. The aim of this review was to describe the importance of sources and neurosurgical approaches for harvesting and preparing brain tissue for cell cultures that are used in laboratory practice for biomaterial testing. Only properly collected tissue can be used for cell culture establishment and then for further testing of biomaterials and this topic is often neglected in experimental practice. Therefore, we have tried to highlight three points in this article: (I) only proper surgical tissue collection can lead to a relevant cell culture; (II) the route of tissue transfer must be clearly defined; and (III) from a biomaterials perspective, the material is transferred from the clinical practice to the preclinical setting to solve a relevant clinical problem.

## 2. The Sources for Brain Cell Isolation

There are many sources of brain cell isolation. Because of interspecies differences, animal cells cannot be directly transferred to study similar processes in humans, although they are readily available, readily accessible, and relatively easy to maintain in culture [26,27,28,29,30,31]. Therefore, human brain cells are desirable. Human sources for such isolates include neonatal and adult brains. The tissue may be normal or neoplastic, depending on the purpose of the isolation [18,32,33].

**Figure 1:**
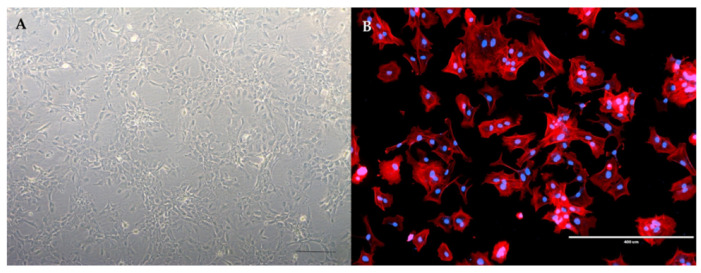
(**A**) The primary culture of human astrocytes in low-density culture. Individual polygonally shaped cells are evident. Images were taken at ×50 magnification on Zeiss Axiovert 40 inverted microscope. Scale bar = 200 µm. (**B**) The immunocytochemical characterization of human astrocytes. The cell morphology was appreciated with orange fluorescent phalloidin conjugate, selectively binding to actin filaments (red). In low-density cultures, astrocytes show a polygonal shape with actin filaments adjacent to the cell membrane. Nuclei were counter-stained with 4′,6-diamidino-2-phenylindole (DAPI) blue. Images were taken at ×10 magnification on EVOS FL fluorescence microscope. Scale bar = 400 µm.

Neuroglia is the most common cell population used in research (Figure 1).

Human neonatal bran is still a good source for cell isolation but is not commonly used because of the difficulties in obtaining the tissue and its further processing [32]. In research in our laboratory, we use only adult brain tissue for astrocyte isolation for several reasons [32,33]. First, according to literature reports, adult astrocytes are more suitable than neonatal astrocytes. It is known that neonatal brain astrocytes have very limited proliferation activity, do not survive long in culture, and cannot be readily subcultured. These cultures are therefore of limited utility [32,34,35]. Furthermore, the differentiation of neonatal cells may be incomplete as they lack normal cell partners or differentiation signals [32,36]. In addition, they may have different gene expression characteristics and are considered more active than mature adult brain cells [37]. The experimental results from neonatal cells cannot be directly transferred to the adult cells [27,30,32]. Therefore, adult brain-derived neuroglial cells provide a valuable and convenient model for experiments, as their pathophysiological mechanisms cannot be equally studied in neonatal culture [27,30,32,33].

**Figure 2:**
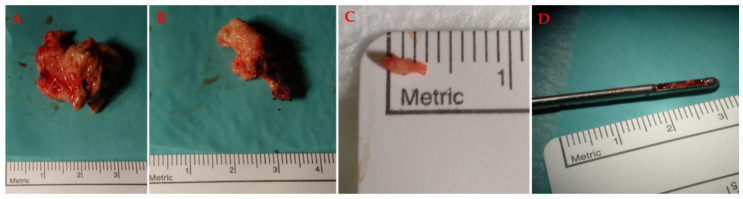
Various resection specimens obtained during brain surgeries. (**A**) In open surgery and in gross resections, abundant tissue is obtained that can be used for further processing in the cell laboratory, as in open glioblastoma surgery. (**B**) The resection specimen of glioma obtained from open biopsy or smaller keyhole approach. (**C**) The glioma sample from the needle biopsy. (**D**) The biopsy needle with the tissue sample. The images were taken during routine neurosurgical procedures and the tissue was also collected for the purpose of cell culture isolation. The ethical approval number was KME RS 0120-565/2020/5.

Secondly, compared to neonatal brains, tissue is much more readily available for isolation in adults, both in terms of quantity and frequency of collection. Neonatal brains can be obtained from foetuses, usually removed at 9 to 12 or 22 weeks of age during elective abortions. Since there are not many elective abortions, a suitable tissue source question arises [32,38]. In addition, the timing of tissue collection is problematic, and there must be a strict collaboration between the clinical department and the laboratory. Furthermore, not all foetuses are suitable for isolation. Only brain-shaped foetuses collected by the surgical procedure of vacuum aspiration can be used. In foetuses where abortion was performed after a medical procedure, the tissue is not suitable because the pharmaceutical agents used to kill the foetus can alter the viability of the cells and thus hinder the development of the primary cell culture [32,38,39]. On the other hand, adult tissue is easily accessible because there are many other surgical procedures that can make the tissue available for experimentation. Normally, the tissue can be collected during gross resection in open surgeries and in different types of biopsies, open and stereotactic (Figure 2).

The most common sources of adult brain tissue are from the cortex of patients undergoing craniotomy for tumour, trauma, vascular, and epilepsy surgery [17,18,26,32]. For tumours, necrotic portions should be avoided, and only viable neoplastic tissue should be harvested. In surgery for an unruptured aneurysm, an elective operation, a tiny amount of brain tissue is removed from the aneurysm dome that is not affected by pathological conditions. In epilepsy surgery, the tissue is abundant and histologically intact. In particular, deep brain structures are accessible in deep brain lesions and brain biopsies, e.g., the hypothalamus, basal ganglia, and insula. All these specimens are excess brain tissue, which is usually small and more delicate, making it prone to desiccation and autolysis if not appropriately stored [32,33,39,40,41]. Some limitations may exist, such as brain trauma when the tissue is removed. Care must be taken to use tissue from the margin of the resected specimen and not to include necrotic and contaminated tissue, as this contains nonviable cells and increases the risk of contamination [17,32]. Similarly, samples taken directly from the penumbra, where the tissue is damaged but still viable, are best avoided. If possible, it must come from the margin of the resected specimen where the tissue is microscopically intact [17,36]. In this way, it is possible to partially regulate the tissue sample’s location and the condition and nature of the cells intended for isolation. Other limitations of traumatized brain tissue include re-expression and modification of cellular markers, changes in protein expression and resulting variations in immunostaining, morphology changes, possible cell damage from extracellular and intracellular oedema induced by the insult, resulting difficulties in cell culture growth, and reduced number of passages [32,42,43]. In addition to trauma, various nervous system disorders such as metabolic disorders (hyperammonemia and hypoglycemia), ischemia, hypoxia, and epileptic seizures are associated with neuroglial swelling. Patients after traumatic brain injury may experience marked changes in extracellular ion concentrations, including decreases in Na^+^, C^1−^, and Ca^2+^, increased K^+^ concentration, decreased extracellular pH, and accumulation of excitatory neurotransmitters, which may be involved in various modifications of function, protein expression and cell morphology [27,44]. For example, adult brain reactive astrocytes, formed in response to various injuries and then plated in culture, re-express some markers of developing astrocytes, including genes for DNA binding, apoptosis, cell cycle regulation, cell adhesion, cytoskeleton, extracellular matrix formation, and signal transduction genes. Usually, adult astrocytes express more genes for metabolic enzymes than neonatal astrocytes [26,37]. The most noticeable morphological change is the swelling of astrocytes, which is reversible and changes the morphology once the cells are placed in culture. Although these effects are mostly pronounced in astrocytes, as they are the most reactive cells, they can also be observed in oligodendrocytes, neurons, and microglia [27,32,38,44].

When comparing neonatal and adult brains, in addition to the age differences of the neonatal donors, there are other factors that can affect cell isolation and increase the variability from case to case, such as the different conditions of the brain tissue before the cultures are prepared, since the age of the foetus can never be determined precisely and varies by one to two weeks.

**Figure 3:**
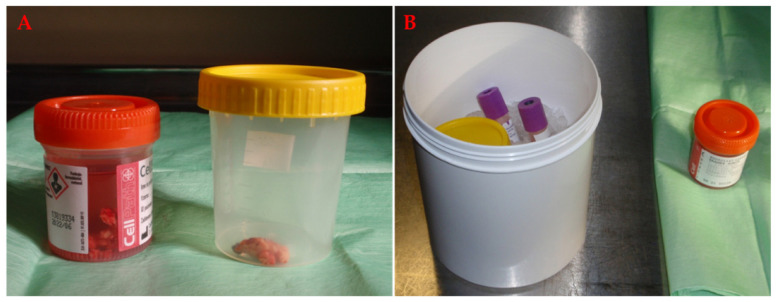
(**A**) Glioblastoma tissue specimen prepared for transport to the cell laboratory for cell isolation and pathology for histopathological evaluation. The sample destined for pathology is preserved in formaldehyde solution (left), and the tissue for the cell laboratory is transported in saline (right). (**B**) The goal is to transport the tissue on ice and as quickly as possible to reduce cell death.

Transport to the laboratory is very important and the time and mode may vary (Figure 3).

The transport to the laboratory may vary and it is usually longer for neonatal brain specimens collected in abortions. The transport time is typically less than two hours [19,45]. In addition, strict ethical rules were established to regulate the use of embryonic stem cells, and therefore the use of foetal tissue was restricted [19,45]. In adults, however, tissue is usually more stable as it is collected during resections and biopsies, and it reaches the laboratory much more quickly. Because adult brain tissue is readily available and abundant due to the greater number of surgeries than neonatal sources, it is the preferred tissue source for experimentation [34,46,47,48,49]. In our clinical department, the tissue transport time is up to 20 min.

## 3. The Challenges of Isolating the Brain Tissue Cells

Isolation of cells from human brain tissue presents many difficulties and challenges. The first step is to collect sufficient and preferably representative tissue, depending on the goal of the isolation. If the goal is to establish cultures of normal human astrocytes, it is necessary to obtain a healthy portion of the brain. Tumour tissue is not suitable for the isolation of astrocytes, nor is the tissue obtained during operations of various cerebral haemorrhages. Brain matter near the hematoma (i.e., the penumbra) or macroscopically damaged brain tissue (in the case of trauma) are not suitable for culture and isolation because they are often necrotic or subvital [32,50]. In addition, the goal of a minimally invasive surgical technique for hematomas and tumours would not allow injury to adjacent brain areas that have been grafted. Tumour tissue can be used to isolate tumour cells, such as in various gliomas [51,52]. All specimens obtained are excess brain tissue. Histopathologic diagnosis should never be abandoned in favour of cell isolation. An adequate amount of tissue is essential for a good isolation result. Special care must be taken not to damage, crush, or coagulate surgically collected specimens, which can sometimes be difficult. This was also discussed in the article. Similarly, samples that are heavily contaminated with blood can be a difficult task for isolation, as the high percentage of red cells can interfere with the isolation process. Similar characteristics are also important in the isolation of other brain cell types [39].

One of the challenges in isolating brain cells is the source of the tissue. Human sources include adult and neonatal brains, both of which are distinctly different [32,39]. Differentiation of neonatal brain cells may be incomplete due to lack of normal differentiation signals and differential gene expression [32,36,37,38]. This may be important when using cell cultures to study neurodegenerative diseases. Experimental results from neonatal cells cannot be directly extrapolated to adults, highlighting the superiority of adult astrocyte and oligodendrocyte cultures in these cases. Factors that may influence brain cell isolation and increase case-to-case variability include age differences in neonatal donors and different donor tissue conditions [27,30,32,33,34]. In addition, the tissue source for adult astrocyte isolation is much more readily available than neonatal brain tissue obtained during elective abortions at 9 to 22 weeks of age [32,38,39]. Further not all foetuses are suitable for tissue collection. Abortion after medical intervention does not provide suitable tissue because the pharmaceutical agents used to kill the foetus can alter cell viability and interfere with cell culture development [39,40]. On the other hand, adult tissue is readily available. There are many more surgical procedures that can provide the tissue for experiments. Usually, the tissue can be collected during gross resection in open surgeries and in different types of biopsies, open and closed. The most common sources of adult brain tissue are the cerebral cortex during craniotomies for tumour, trauma, vascular, and epilepsy surgeries. In particular, deep brain structures are accessible in deep-seated brain lesions and in brain biopsies, for example, the hypothalamus, basal ganglia, and insula. This is possible through the use of sophisticated imaging and surgical techniques, and cells from these areas have different properties than cells from cortical and subcortical regions [32,33,39,40,41]. In our opinion, adult brain tissue is readily available and abundant due to the greater number of surgeries compared to neonatal sources, making the former a preferred tissue source for experimentation. The timing of tissue collection is also important and there must be strict collaboration between the clinical department and the laboratory to limit the deterioration of the collected tissue. Transport to the laboratory can vary and is usually longer for brain samples taken in abortions. In adults, the tissue is usually more stable as it is collected during the biopsy and reaches the laboratory much more quickly [19,32,36,45].

Other, factors that affect brain cell isolation and may increase case-to-case variability include age differences in neonatal donors (the age of the foetus can never be accurately determined and varies by one to two weeks) and different conditions for donor brain tissue prior to culture. Neonatal brain cells differ from those isolated from adults, and differentiation of neonatal brain cells may be incomplete because normal cell partners or differentiation signals are absent [32,36]. In addition, neonatal brain cells have different gene expression characteristics and are considered more active than adult cells, which are considered mature [37]. The number of genes expressed in cultured adult and cultured neonatal cells is comparable, but there are differences in gene classes. For example, adult astrocytes express more genes for proteases and protease inhibitors than neonatal astrocytes. More genes for metabolic enzymes are expressed in adult astrocytes than in neonatal astrocytes, suggesting a higher level of metabolic activity. In contrast, neonatal astrocytes express more active genes for DNA binding, apoptosis, cell cycle regulation, cell adhesion, cytoskeleton and extracellular matrix formation, and genes for signal transduction. Only astrocytes in postnatal brains were found to express the glial fibrillary acidic protein (GFAP) gene [26,37,53]. All this is important when using cell cultures to study neurodegenerative diseases. The experimental results from neonatal cells cannot be directly transferred to the adult cells, and this is where the superiority of adult astrocyte culture for studying neurodegenerative diseases becomes apparent [27,30,32,33,34].

Once a suitable tissue is available, properly collected, stored, and transferred to the laboratory, an appropriate isolation technique must be developed, which differs depending on the cell type. The goal is for this technique to be rapid, simple, inexpensive, and produce sufficient quantities of isolated cells that exhibit specific biochemical and physiological properties [31,54]. During isolation, it is necessary to develop an effective technique for maintaining the isolated cells, which is often challenging [19,31,32]. Despite ideal properties untransformed cell culture can dedifferentiate and cells lose their phenotypic properties after a certain number of passages. This can be somewhat corrected with special cultivation conditions and selective cell media [31,32,50,55].

In cell isolation, there is always the possibility that the cells in culture are not the target cells. When preparing a pure cell isolation from the brain, other unwanted contaminating cells may be present (e.g., during astrocyte isolation, microglia and oligodendroglia are potential contaminants) [26,36]. The isolation procedure for a particular cell type is specific. For example, isolation of astrocytes is relatively easier and faster than isolation of oligodendrocytes (or oligodendrocyte progenitor cells (OPCs)). First, the proportion of astrocytes in the brain is higher compared to oligodendrocytes, making them the most important cells in the mixed primary cell culture [26,27,34,35,44,56,57,58]. Astrocyte cultures generally contain less than 5% microglia and an insignificant number of neurons or oligodendrocytes [36]. Second, the protocol for isolating oligodendrocytes is very different from that for astrocytes and is more difficult than culturing the other cell types. In mixed glia cultures from brain tissue, astrocytes and oligodendrocytes are often obtained by establishing mixed glia cultures [26,59]. Protocols for the isolation of oligodendrocytes have been described elsewhere [36,45,46]. When plating a primary culture, it is relatively easy to obtain pure preparations of astrocytes with more than 95% homogeneity. Within about a week, the astrocytes proliferate and grow in a monolayer. The culture flasks contain a monolayer of astrocytes and few oligodendrocytes scattered on the surface [46]. Thus, the oligodendrocytes and OPCs are removed during medium changes, washing of the culture, and subsequent passages. Astrocytes proliferate faster than oligodendrocytes and the cells that are in the cell culture minority are lost from the culture during the growth of the main cells. Third, oligodendrocytes in culture are completely different from astrocytes in size, shape, morphology, and branching of cell processes and it is possible to distinguish one cell type from the other under the microscope [26,36,45,46]. Fourth, the markers in oligodendrocytes are very different from those in astrocytes [26,36,42,43,54,56,60].

In the human brain, microglia are present in all regions and account for a large proportion of the total cellular composition of the brain, estimated to be as high as 12% [61]. Although brain cell cultures are relatively easy to prepare, they are often contaminated by microglial cells. Microglia are separated by mechanical separation taking advantage of the physical properties of these cells [26,32,33,44,62,63]. It is possible to reduce the amount of microglia to as low as 5% or less, and according to some authors even to less than 1% [36,64,65].

Challenges and limitations in collecting tissue samples for cell culture research include technical obstacles and limitations primarily related to surgical procedures. These include accessibility of deep brain structures, technical limitations of instruments, and last but not least, surgical skills. Good collaboration between the clinical department and the cell laboratory is essential for good timing, appropriate tissue storage, and smooth tissue transfer [30,34,58,66]. An appropriate isolation technique specific to the brain cell type is a matter of trial and error and it may take a long time to prepare a suitable cell culture [33,50,67,68].

In vitro brain cell cultures can include various cell types, such as astrocytes, oligodendrocytes, microglia, microvascular endothelial cells, neurons, ependymal cells, and oligodendrocyte progenitor cells (OPCs). Key advantages of brain cell cultures include the ability to perform biochemical analyses of individual identified cell types, reduced cell complexity (compared to whole brain), the ability to fully control the cellular environment, imaging and electrophysiology of individual cells, co-culture capabilities, and manipulation of gene expression [36]. Moreover, cultured brain cells from different regions are heterogeneous in their expression of immunoreactive surface markers, chemokines, and cytokines and differ in their morphology. Therefore, it is more useful to study these cells separately under in vitro conditions [28,36,44,57].

The next stage of functional cell models using cell cultures are human mini-organs or the so-called organoids. Their establishment is one of the greatest scientific advances in regenerative medicine, especially in the use of brain cells. Organoid technology is based on classical three-dimensional culture techniques that support the cell-autonomous self-discovery reactions of stem cells to create micrometre- to millimetre-sized versions that correspond to human organs. This organoid technology is still in its infancy and far from clinical application. However, it is expected to open up new possibilities and change the way we do transplantation and organoid research [69,70]. Here we see the ultimate goal of cell cultivation, the development of new isolation techniques and cell models. Again, the foundation is tissue collection, which determines the success of cell isolation.

## 4. A Brief Description for a Culture Protocol for Brain Cells

There are many potential sources of brain tissue, from different species, each with their advantages and disadvantages [56,71]. There are few reports on the isolation of human astrocytes, and these cells do not readily grow in culture [34]. Their isolation and maintenance is therefore difficult [27,28,34]. The main advantages of isolated astrocyte cell culture are the ability to fully control the cellular environment, co-culture experiments and manipulation of gene expression, imaging and electrophysiology of individual cells, and the ability to perform biochemical analyses of individual identified cell types [39,40,41,45]. During the isolation process, it is first necessary to collect an appropriate tissue sample as effectively as possible to prevent necrobiotic processes, as described previously, and then to develop an effective cell isolation technique for preserving the isolated cells [34,47]. The isolation technique for human astrocytes is briefly described below.

### 4.1. The Source of the Tissue

Tissue for human astrocyte isolation is obtained during various cranial surgeries, most commonly epilepsy surgery, vascular surgery (arteriovenous malformations and aneurysms), and brain necrectomy in adult neurotrauma patients. Permission to use human brain tissue is essential. In our laboratory practise, we have obtained approval from the ethical committee and obtained written informed consent from the patient or their relatives (our ethical approval number is KME/98/14). Brain tissue is collected under sterile conditions from cortical and subcortical regions, depending on the surgery performed. When possible, both the grey matter and the underlying white matter are removed during surgery. After surgical resection, the fragments of viable tissue are collected and placed in cell medium or saline to prevent desiccation and immediately taken to the laboratory. We use Advanced Dulbecco’s Modified Eagle Medium (DMEM), supplemented with 100 IU/mL penicillin, 0.1 mg/mL streptomycin, and 2 mM L-glutamine.

### 4.2. Preparation of Tissue for Cell Culture

Tissue fragments are washed in phosphate buffer solution (PBS) containing penicillin and streptomycin and cell culture medium is added. The tissue is first cut into small pieces to achieve coarse mechanical decomposition and additionally resuspended by pipetting. This is followed by centrifugation at 300× *g* for 15 min. The cell sediment is harvested, washed with Advanced DMEM and centrifuged. The sediment is resuspended again in cell culture medium containing antibiotics and foetal bovine serum (FBS) and plated out into tissue culture flasks. The resulting cell suspension is incubated at 37 °C and 5% CO_2_ for approximately one month, resulting in preferential proliferation and survival of astroglial cells. The medium is normally changed twice a week. Contaminated cells such as microglia can be removed when the medium is replaced, by washing the culture with the medium and removing the loosely attached cells. Once a confluent monolayer of flat cells has been obtained, the purity of the cultures can be assessed.

### 4.3. The Culture of Primary Astrocytes

Primary astrocytes are cultured in culture flasks and incubated at 37 °C in a controlled atmosphere with 5% CO_2_. After one month in culture, they are 100% confluent and are cleaved with trypsin, usually at a ratio of 1:3. After centrifugation, the cell sediment is resuspended in fresh medium with FBS and transferred to cell culture flasks. In this way, the cell culture of the first passage is obtained. The cultures are then incubated again for one week until they are 95% confluent.

Phenotypic and functional characterization of the cultured cells is performed using immunocytochemistry and looking for the presence of the major astrocyte markers. Among the most common are GFAP, protein S100B, and glutamate aspartate transporter (GLAST). Other popular markers for astrocytes include glutathione peroxidase, GLT-1 (EAAT2 in humans), glutamate transporter, glutamine synthetase, ALDH1L1, and the astrocyte-specific water channel aquaporin 4 (AQP4). Cell morphology can be studied with actin cytoskeleton labelling. Immunocytochemical techniques allow the detection of specific molecular markers in astrocytes and are essential tools for the identification and characterization of the isolated cells [26,29,33,35,56,71].

## 5. Brain Cells and Biomaterial Development

Both tissue engineering and biomaterial development for central nervous system regeneration are the focus of research [72,73]. In vitro studies have shown promising results in this area [72,73]. Biomaterials used in these applications have demonstrated numerous functions, such as neuroprotection, induction of axonal regeneration, modulation of immune responses, and participation in healing after injury [74,75]. In addition, biomaterials can be designed to facilitate and guide axonal spreading during regenerative phases and potentially be used for axonal renewal after injuries of different aetiologies [76,77,78]. Since the brain microenvironment plays an important role in brain cell function and structure, these interactions must also be considered in the development of the biomaterial. Its composition should be comparable to the composition of the extracellular matrix, which includes proteoglycans, hyaluronic acid, laminin, and tenascins, all of which have an important influence on the growth of brain cells, not only neurons. When culturing brain cells for biomaterials research, this is crucial in order for them to maintain an optimal cell phenotype. Therefore, the physiological morphology of brain cells in culture can be achieved by culturing these cells in a three-dimensional matrix that provides structural support and the proper extracellular matrix proteins [30,32,44]. Therefore, the new biomaterials must stimulate the cell phenotype that promotes axonal regeneration and neuronal survival [30,44].

To determine the response of brain cells to physiological and pathophysiological conditions, in vitro experiments are performed in cell models and combined with different biomaterials. It is therefore crucial to develop biomaterials that interact with brain cells in an appropriate manner. In these in vitro studies, numerous cellular events such as cell proliferation, migration, adhesion and morphological changes, brain cell growth, and gene and protein expression can be determined [32,44]. The most popular biomaterials in such studies include collagen gels, hyaluronic acid-based hydrogels, combinations of gels with collagen and hyaluronic acid, combined gels with variable proportions of collagen, hyaluronic acid, and matrigel, polymer scaffolds, and patterned substrates [44,56,79].

In various pathological conditions of the brain and spinal cord, not only neurons are affected. Many other cells with supporting and protective functions, such as astrocytes, oligodendrocytes, microglia, and endothelial cells, can also enter the pathological circuits [80,81]. As a result of the irreversible loss of neurons and the limited ability of the central nervous system to cope with the damage, these diseases often lead to long-lasting neurological deficits [82,83,84]. In order to limit the extent of neuronal damage and promote recovery of injured brain and spinal cord areas, the focus is on limiting the region of the penumbra and promoting regeneration of central nervous system cells. In bioengineering, cell-based approaches have been used extensively to overcome the effects of glial scarring and replenish the lost cells, especially neurons. The idea of bioengineering bridging materials such as hydrogels and conducting structures is to promote neuron regeneration, enable targeted reconstruction, replace neuronal circuits, limit glial scar formation, and promote integration with host cells [81,85,86]. Such biocompatible materials that promote glial cell attachment and migration will be important for future repair strategies for injured neural tissue [80,85,87].

## 6. Relationships between Tissue Sampling and Biomaterial Testing

Biomaterials research aims to understand the biological response of tissues and organisms to artificial implants. This has recently allowed great progress in the development of artificial materials. Research into the influence of biomaterials on the human body and vice versa begins with in vitro studies, which are particularly important in the development of biomaterials. Each biomaterial is developed for its specific use. In vitro testing is followed by testing under in vivo conditions, which may progress to clinical research [88,89,90].

The main goal in biomaterials science is to develop materials that react specifically with the biological environment for which they are designed [91,92]. This is a so-called tissue regeneration approach. The biomaterials can serve as temporary scaffolds or cell anchorage sites for three-dimensional tissue structures that are colonized by specific cell types and enhance tissue regeneration [91,93,94]. Functional cell models contain one or more specific cell cultures and biomaterials in a specific experimental environment. The tissue, which is usually collected during surgical procedures, is the basis for the development of an appropriate cell culture. If the tissue has not been properly collected or necrobiotic processes have occurred, cell isolation will not be successful, or the culture will be suboptimal. This will also affect the experimental conditions and the test results of the biomaterials. It is therefore essential that the surgical procedure is suitable for tissue uptake [93,94].

Some biomaterials are degradable over time within the tissue in which they are implanted, whereas others are permanent. A novel and specific function of biomaterials is their use in molecular transfer into target tissues for the treatment of disease. An example of this is the transfer of encapsulated genes into the cells of diseased tissue [95,96,97,98].

The goal of analytical evaluation of the biomaterial is to determine the presence of its beneficial responses, including integration into host tissues and possible resorption of the biomaterial if required, and to evaluate adverse effects. The ultimate goal is to determine the biocompatibility or safety of an implant in a given clinical setting [95,96]. Specific parameters that are important for biomaterial evaluation include biocompatibility studies first. This is followed by other assessments, such as evaluating the presence, extent, and characterization of: (I) inflammation, including the extent, cellular composition, and distribution, (II) degenerative changes in peri-implant tissues, (III) fibrosis, which may include interstitial/dissecting fibrosis and the presence of a fibrous capsule, (IV) the extent and composition of tissue ingrowth, particularly into porous and resorbable implant materials, and (V) implant material-specific parameters such as integrity, debris, and fragmentation, as well as the location of degraded biomaterial [99,100].

Biological responses to biomaterials are evaluated by characterizing and quantifying these responses macroscopically and microscopically. Pathological assessment includes the presence of necrosis, cell metaplasia, protein exudation, and ossification. Histological assessment provides information on the specific components of the biomaterials. Resorbable biomaterials are usually associated with some degree of inflammatory cellular accumulation, neovascularization, and fibrosis. Regarding the specific cellular responses, inflammation is an important parameter, and the assessment should include both the overall degree of infiltrates and the amount of cellular components. Localization of inflammatory infiltrates may also provide important information about different responses to different implant components [99,100]. Other implant-associated parameters are particularly important in determining implant integration. These include neovascularization, the presence of fibroblasts, and the deposition of extracellular matrix [94,96,100].

## 7. Neurosurgical Approaches for Brain Tissue Sampling

The use of new technological achievements and the number of invasive procedures in medicine are increasing [48,49]. With the advent of new technological capabilities that support the development of neuroendoscopic and neuronavigational techniques and instruments, surgeries are also becoming less invasive for patients [101]. In addition, researchers believe that basic research can also benefit from these new approaches. They provide the opportunity to collect brain tissue samples from an increasing number of neurosurgical pathologies at multiple sites that can now be accessed with minimal potential morbidity, faster, easier, less invasive, more frequently, and with less tissue damage, contributing to higher cell yields for isolation in the laboratory [102,103,104].

**Figure 4:**
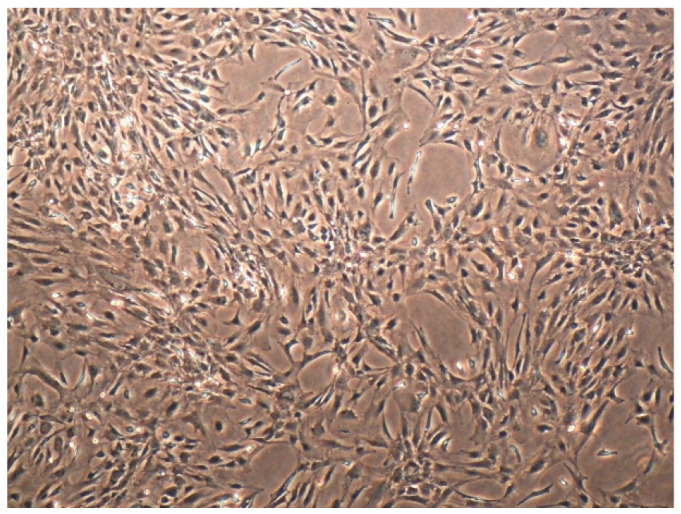
The culture of glioblastoma cells isolated from the resection specimen. A characteristic polymorphic cell appearance with sparse cytoplasm and differently shaped nuclei are seen. Nicon Diaphot 300 inverted microscope. Scale bar = 100 µm.

When considering brain surgery, it is essential to recognize that the primary goals of surgery vary depending on the pathology [105]. These include relieving the increased intracranial pressure for various reasons, treating the vascular pathology or haemorrhage, evacuating the hematoma, reducing the tumour as safely as possible, obtaining tissue for histologic diagnosis, and repairing the head injury [106]. Secondly, not all pathologies are appropriate for tissue collection. Isolation of non-transformed brain cells requires obtaining a healthy portion of the brain. Tumour tissue is not suitable for such isolation, nor is tissue obtained during surgery of various brain haemorrhages. The brain matter surrounding the hematoma (i.e., penumbra) is unsuitable for culture formation and isolation processes because it is often necrotic or nonviable [17,32,36]. On the other hand, tumour tissue from astrocytomas, oligodendrogliomas, and glioblastomas is used to isolate neoplastic cells [106,107] (Figure 4).

It is also important to note that the treatment goal, which includes preserving the patient’s neurological function, always comes first, and the tissue for cell isolation comes second. Thus, all surgical specimens obtained are excess brain tissue that will not be used for further diagnostics. Additionally, ethical approval and informed consent must be obtained from the patient and family before any experimental manipulation with the tissue [108].

Numerous neurosurgical approaches used in clinical practice provide a welcome source of healthy and diseased brain tissue [104,105,106]. In recent decades, neurosurgeons have developed and refined surgical techniques that make operations less invasive and more efficient, optimize surgical outcomes, and help limit the potential for neurologic morbidity [106]. Three-dimensional (3D) neurosurgical planning, the use of augmented reality in neuronavigation, neuromonitoring, direct cortical and subcortical stimulation, corticography, diffusion tensor imaging (DTI), functional magnetic resonance imaging (fMRI), tumour fluorescence (5-ALA), and awake brain surgery are some of the modern workhorses in performing the least invasive, most effective and maximally safe resections. The interaction of sophisticated surgical microscopes and neuronavigational systems brings the principles of robotics to the image-guided resection of tumours. The procedures can be performed under general anaesthesia or in a scalp block as in awake surgeries [106,107,108,109,110,111]. The type of neurosurgical procedure depends on many factors, such as the location and size of the tumour, its vascularity and composition, the diversity of tumours (solitary, multiple metastases, or involvement of many lobes), accessibility, eloquent areas of the brain, the clinical condition and wishes of the patient, and yet the surgical equipment [106]. Of course, as technology advances and surgical capabilities increase, so do the possibilities of obtaining an ideal tissue sample. The most commonly performed neurosurgical techniques are described below.

### 7.1. Open Surgery

**Figure 5:**
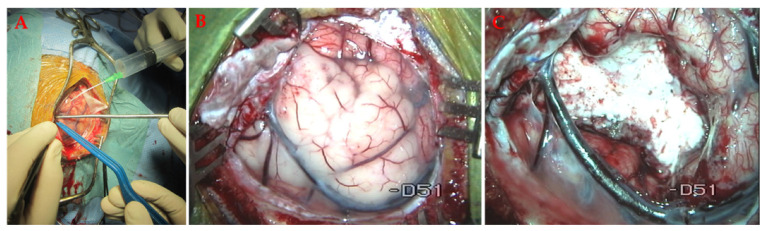
(**A**) Brain exposure in open glioma surgery. (**B**) The primary brain tumour is clearly visible on the surface. The tumour tissue is more swollen and tan white. (**C**) The resection cavity after tumour removal. The drainage vein below the tumour was left in place because it is essential to maintain normal brain drainage. All images were taken during routine neurosurgical procedures at our medical centre with patient and ethical committee approval.

Open surgery with its modifications is one of the most commonly performed procedures for primary and metastatic brain tumours, for all traumatic brain injuries, and most vascular pathologies (Figure 5).

Open surgery offers the best chance of improved survival [108]. It is used for tumours located in non-eloquent areas where maximal resection of the tumour is desired. It provides good accessibility, visibility, and debulking of large tumours, thereby relieving intracranial pressure and improving neurological symptoms. The operating microscope offers the best possible view, and micro-neurosurgical instruments must always be available [106]. Superficial lesions are particularly suitable for resection, as are highly vascularized lesions where stereotactic techniques are contraindicated [112]. In the vicinity of eloquent areas, particularly sensory and motor areas, intraoperative electrocorticography and cortical mapping can be used to limit potential surgical damage. Tumour removal by open surgery also provides information about the extent of involvement of nearby structures and helps reduce sampling errors during a stereotactic biopsy. In vascular pathology and trauma cases, open surgery provides good access to the severed vessel and the opportunity for brain decompression or necropsy [105,106,113]. The open approach means that the tissue samples taken are usually plentiful. Therefore, the tissue can be used liberally for cell isolations, separating the nonviable parts and using only the most appropriate ones.

A standard open approach involves a linear or curvilinear incision to provide direct access to the cortex or tumour, depending on its location [114]. Typical neurosurgical techniques include pterional, bifrontal, convex, interhemispheric, and suboccipital craniotomies. Less commonly, orbitozygomatic and transsphenoidal craniotomies are used, which provide better access to the skull base. Decompressive craniotomy for traumatic brain injury is a special entity that offers great exposure of brain areas, possible necrectomy in damaged areas, and relief of refractory increased intracranial pressure [113,115]. Craniotomy for superficial tumours typically encompasses the entire tumour area, whereas deep tumours can be reached through smaller craniotomies as the intracranial surgical field increases with a larger distance from the cranial bone surface [114]. The working corridor through the brain parenchyma must be maintained with retractors, either spatulas or intermittent retraction with the hand-held instruments during bimanual manipulation of the tumour [104,116]. Transcortical access to the lesion is replaced by trans-sulcal or trans-fissure whenever possible to shorten the corridor. The eloquent areas on the surface and subcortical tracts and vessels are examined preoperatively by digital tractography, fMRI, and high-resolution MRI to obtain a reliable vector for the working corridor [104,106,116]. Care must be taken to protect the vasculature and adjacent normal brain from retraction injury and direct brain traction away from eloquent areas [117]. In severe traumatic brain injury, necrectomy must be minimized, and a reasonable balance must be struck between removal and preservation of structures [118].

Surgical complication rates range from 2% to 9%. Although most patients show improvement in their performance status postoperatively, surgical morbidity after tumour resection is reported to be 8% to 11%. Additionally reported are a 1.5% mortality rate, a 1.5% wound infection rate, and a 4.5% surgical site bleeding rate [119,120]. Postoperative medical complications may occur in 3% to 9% of patients. Of course, trauma patients and those with vascular emergencies have a higher risk of complications, depending on the clinical presentation on admission and the extent of brain insults [108,120].

### 7.2. Keyhole Approaches

**Figure 6:**
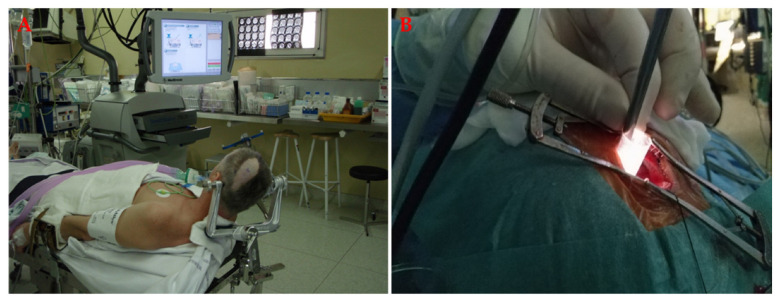
Keyhole craniotomy for primary tumours, which is a modification of the conventional craniotomy. (**A**) The patient is positioned, and the neuronavigation is prepared for guidance. Because less anatomy is visible on the surface, image guidance is essential. (**B**) The insertion of the instruments for tumour resection through the keyhole craniotomy. With this technique, the morbidity associated with access is minimized, as is the complication rate.

A craniotomy can also be performed through a very small craniotomy or a so-called keyhole approach and is a modification of conventional craniotomy [121]. It is particularly suitable for minimizing the morbidity associated with access, especially in primary tumours (Figure 6).

The complication rate here is lower compared to open surgery. Unlike classical craniotomies, keyhole craniotomies are much smaller than the lesions [121,122]. Nevertheless, they allow the local anatomy to be seen and are particularly useful for deep-seated tumours. Because less anatomy is visible at the surface, image guidance is usually required, which increases surgical safety [109]. Keyhole craniotomy has been used effectively and safely to perform both biopsy and gross total resection for numerous primary and secondary brain tumours, with rates of gross complete resection ranging from 74% to 87% [121,122]. However, they are not suitable for the surgical treatment of brain trauma and oedema due to limited accessibility and small exposed areas. The simultaneous use of an endoscope (endoscope-assisted microsurgery) can improve the extent of resection by providing access to the residual tumour in the lateral areas of the resection cavity that are not visible with a standard surgical microscope [123]. As with open surgery, re-navigation can be combined to determine the shortest and most optimal surgical trajectory to the lesion. However, navigation systems do not consider anatomical changes due to brain displacement caused by cerebrospinal fluid loss, tumour resection, osmosis, or manipulation of normal brain tissue. Intraoperative ultrasound or intraoperative MRI may help in brain shift occurring during surgery and in detecting residual tumour [110,124]. In emergencies, a neuronavigational setup may be omitted, especially if the time required for preparation may prolong the surgical procedure. As in open surgery, the tissue samples are generous, especially in gross total resections. Of course, location, nature, and accessibility of the tumour are the factors that influence the amount of tissue and thus its availability for the laboratory. The abundance of tissue available in both open and keyhole approaches provide an opportunity to separate the damaged and necrotic or blood-contaminated parts and to remove only the most appropriate tissue for cell isolation. This can sometimes be done in conjunction with the pathologist who is present in the operating room during surgery. This ensures that the most representative parts are taken for pathology and the most suitable for cell isolation [121,125].

### 7.3. Stereotactic Needle Biopsy

**Figure 7:**
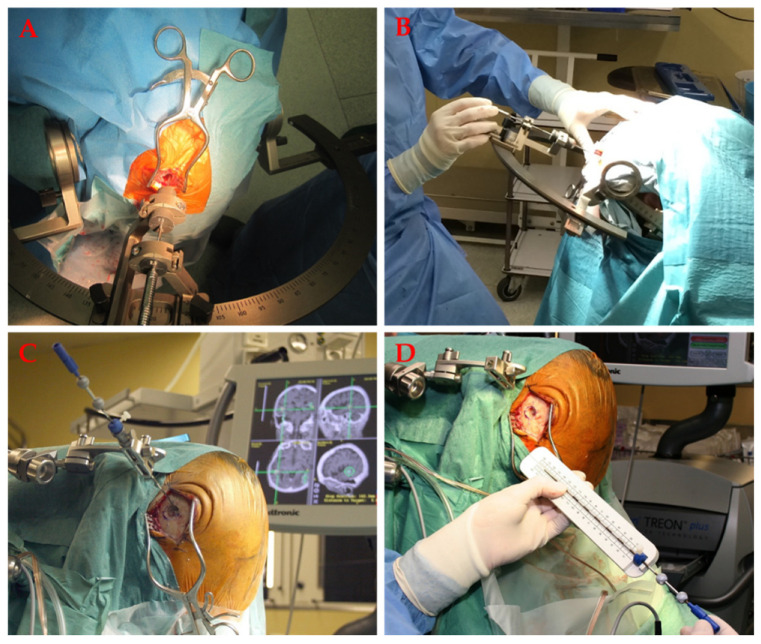
(**A**) Frame-assisted stereotactic biopsy for deep-seated brain lesions. The stereotactic arch with the attached biopsy guidance introductor is visible. (**B**) The insertion of the biopsy needle. (**C**) Frameless stereotactic biopsy. The trajectory is adjusted during the procedure according to neuronavigational panning. (**D**) The biopsy needle for frameless stereotactic biopsy and needle length adjustment.

Stereotactic biopsy is a relatively new technique, first introduced into clinical practice in the 1970s [126]. The aim is to target a minimal area or volume in the brain using a predefined minimally invasive trajectory. The target location is determined according to the reference system composed of various extra- and intracranial markers [127]. A stereotactic biopsy can be performed with or without placement of the stereotactic frame, which serves as an external reference and coordinate system (Figure 7).

Frameless stereotactic biopsy requires preoperative computed tomography (CT) or MRI of the patient’s head with the frame mounted, and image merged. On the other hand, frameless systems (image-guided biopsy) are becoming more popular because their use is more straightforward, faster, and requires only preoperative imaging, usually by MRI. A computer-generated 3D model of the patient’s head and brain (from MRI or CT scans) is matched with the patient’s actual head position by surface registration of the orbitonasofrontal area in the anesthetized patient, which serves as a patient-specific reference. However, some deep-lying lesions in the brainstem and posterior fossa can only be reached accurately and safely with the frame-based cranial systems [127,128,129,130].

A stereotactic biopsy can provide an accurate pathological diagnosis with a 2 mm to 4 mm targeting accuracy. The diagnostic accuracy is high, ranging from 70% to 93% [130,131]. It can be increased by intraoperative use of fluorescein, which increases the diagnostic yield and improves safety. A neurologically intact patient with a small, deeply located solitary or multiple lesion with minimal mass effect is a good candidate for such management. Patients with cystic tumours can be drained by aspiration through the stereotactic needle. Highly vascularized lesions are not suitable for stereotactic biopsy. It should be noted that stereotactic biopsy can only be used for tumours and other neurodegenerative lesions and is a diagnostic rather than a curative procedure [132,133,134,135]. Mortality is about 2% and surgical morbidity is about 3% [136]. 

The tissue samples taken here are small, usually about 1 mm wide and 2 mm or 3 mm long, and can sometimes be heavily contaminated with blood. The size and composition of the tissue sample plays an important role in cell isolation, as it can affect the number and growth characteristics of the isolated cells. Small tissue samples can also lead to sampling errors due to heterogeneity of the tissue [137].

### 7.4. Neuroendoport Surgery

**Figure 8:**
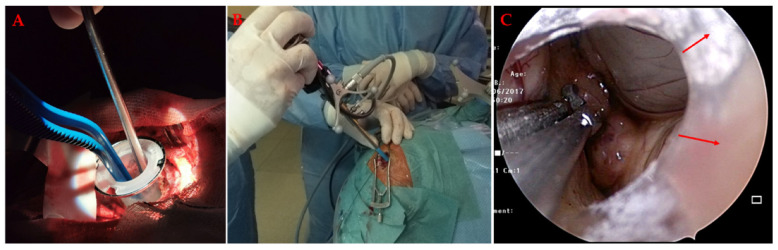
(**A**) The neuroendoport in the position that provides a tubular corridor through the brain to deep-seated lesions. The instruments, the bipolar and aspirator, are then inserted through the neuroendoport into the lesion, with visualization through the operating microscope. (**B**) Alternatively, the endoscope can be used, which is inserted here through the expandable neuroendoport. The neurosurgical instruments follow next. (**C**) Intraventricular tumour resection through the expandable neuroendoport. The arrows indicate the lower edge of the neuroendoport corridor.

The neuroendoport is a cylindrical or tubular retractor system used as a corridor to deep-seated brain lesions (Figure 8).

The neuroendoport can be fixed or expandable and inserted under the operating microscope or with the endoscope [138,139,140]. The neuroendoport allows bimanual surgery with microsurgical instruments under endoscopic or microscopic view, enabling removal of deep lesions with microsurgical techniques. It is particularly valuable for obtaining the least traumatic access to deep brain lesions located in the ventricles, basal ganglia, posterior thalamus, and pulvinar. Prior to the use of neuroendoport, such deep lesions could only be accessed by stereotactic needle biopsy or with the help of retractor systems and neuronavigation [117,139]. Although blade retractors provide good visualization of the surgical field, they can exert pressure on the brain parenchyma and lead to hemorrhage and ischemia caused by vascular injury. The advantages of neuroendoport include more uniform pressure applied to the walls of the surgical corridor, minimizing retractor-induced injury [117,138]. In so-called parafascicular approaches to deep-seated lesions, it is even possible to cut the nerve fibres with the neuroendoport suregy while causing minimal damage to the parenchyma. Modern neuronavigation systems and diagnostic imaging have improved the precision in targeting these lesions and surgical safety [141].

The neuroendoport technique has become the standard of care for resection of astrocytomas, glioblastomas, ependymomas and papilomas, neurocytomas, gangliogliomas, cavernous angiomas, brain abscesses, intraparenchymal hematomas, massive hematocephalus, intraventricular meningiomas, metastasis, colloid cysts, and choroidal arteriovenous malformations [142,143,144]. Early reports have shown that minimally invasive endoscopically guided surgery via the endoport is effective and safe [143,144,145]. Resection specimens are usually well preserved and abundant in microscopic or endoscopically guided neuroendoport surgery, providing a sufficient amount of tissue for cell isolation, which is usually well preserved and viable [24,141].

### 7.5. Neuroendoscopic Surgery

**Figure 9:**
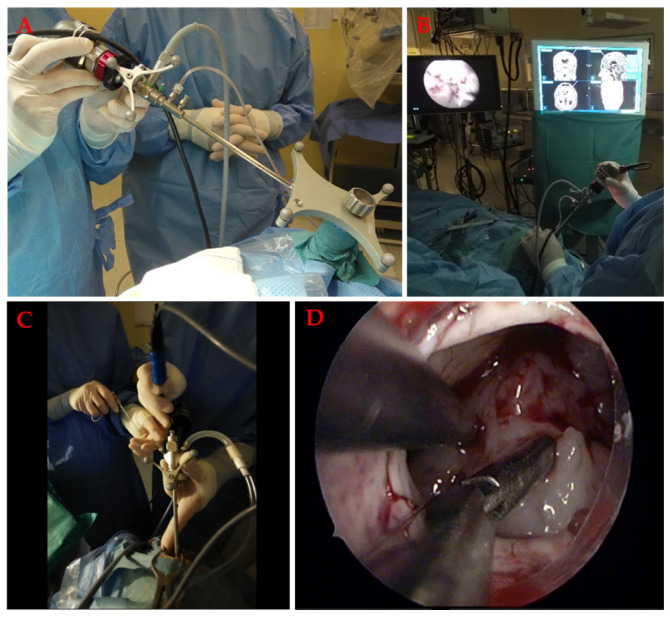
Neuroendoscopy. (**A**) The neuroendocoscope is first navigated. (**B**) During the procedure, the surgical field is observed on the monitor. The exact position of the tip is controlled by a second monitor, which is coupled with the neuronavigation system. (**C**) Full endoscopy with two working channels. (**D**) Endoscopic view during tumour resection.

Neuroendoscopy (Figure 9) involves the use of endoscope to treat various pathologies of the central nervous system.

The technique dates back to the early 20th century and has significantly evolved since then [146,147]. In the beginning, neuroendoscopic procedures were limited to the ventricles (ventriculostomy). Today, however, navigated neuroendoscopy is used to treat a wide range of intracranial pathologies in and outside the ventricles, including biopsy, resection of intraventricular lesions such as colloid cysts and small avascular tumours, intraparenchymal tumour biopsy or resection, resection of the sellar, midline, anterior skull base, and pineal region tumours, cyst or abscess evacuation, cyst fenestration, implantation of radioactive seeds, marsupialisation, endoscopic suturectomy in scaphocephaly, and as an adjunct to microscope-used procedures [142,147,148,149,150,151,152].

Neuroendoscopy is considered a minimally invasive technique with the aim of reducing attachment related brain trauma and improving visualisation of the tissue through better magnification and illumination [147,153]. Skin wound, craniotomy, and brain exposure are minimal, as is brain retraction. Full-endoscopic surgery can be effectively used for ventricular pathologies such as tumour biopsy, removal of hematomas, cystic lesions, endoscopic third ventricusostomy, septotomy, aqueductoplasty, or partial tumour removal (in combination with the endoscopic continuous aspiration device- CUSA) [150,151,152]. Access is gained through the working channels of the neuroendoscope, usually one or two. The simultaneous use of two instruments allows some tissue manipulation. In endoscope-assisted surgery, on the other hand, the neuroendoscope is used only as a visual aid instead of the surgical microscope. The instruments are positioned to the side of the endoscope, and bimanual manipulation (microsurgical technique) is possible. It is inserted through the neuroendoport or natural passages (nasal cavity) and provides all around visualization and access to previously inaccessible tumours, the skull base, and deep neural structures and vessels [150,154]. Neuronavigation can be used in conjunction with neuroendoscopy to select the optimal burr hole or neuroendoport position and to choose the safest trajectory to the lesion, reducing the risk of damage to vital structures [140,142,147]. Furthermore, in endoscope-assisted surgery, the endoscope can be used as an adjunct to traditional microscopic surgery for final inspection of the resection cavity, as it provides an oblique view [148,155].

Tissue specimens obtained during neuroendoscopy vary in size depending on the type of neuroendoscopic approach, i.e., full-endoscopic, or endoscopically assisted. Samples obtained by the latter technique are richer and better preserved because tissue fragments collected by the full-endoscopic method must fit into instruments that slide through the narrow neuroendoscopic working channel and are therefore small and often crushed. This can sometimes hinder successful cell isolation as the number of necrotic cells. Care must be taken not to over-coagulate and crush the tissue with the endoscopic forceps as this can lead to necrosis of the cells in the samples [154,156].

## 8. Conclusions

New technological advances in brain surgery have not only brought relief and better treatment options to patients, but also to researchers by facilitating the collection of tissue samples from different sites of the brain and with increasing precision, tissue quantity and preservation. The type of neurosurgical procedure depends on many tumour- and patient-related factors and indirectly affects the quality of the harvested tissue, which is essential not only for the final diagnosis and treatment but also for the new research opportunities. Since the tissue sample forms the basis for further tissue processing, its integrity and condition are critical to isolation success.

## Data Availability

The data presented in this study are available on request from the corresponding author.
